# Helically agitated mixing in dry dilute acid pretreatment enhances the bioconversion of corn stover into ethanol

**DOI:** 10.1186/1754-6834-7-1

**Published:** 2014-01-03

**Authors:** Yanqing He, Longping Zhang, Jian Zhang, Jie Bao

**Affiliations:** 1 State Key Laboratory of Bioreactor Engineering, East China University of Science and Technology, 130 Meilong Road, Shanghai 200237, China

**Keywords:** Dry dilute acid pretreatment, Helically agitated mixing, CFD modeling, Simultaneous saccharification and fermentation (SSF), Corn stover, Ethanol

## Abstract

**Background:**

Dry dilute acid pretreatment at extremely high solids loading of lignocellulose materials demonstrated promising advantages of no waste water generation, less sugar loss, and low steam consumption while maintaining high hydrolysis yield. However, the routine pretreatment reactor without mixing apparatus was found not suitable for dry pretreatment operation because of poor mixing and mass transfer. In this study, helically agitated mixing was introduced into the dry dilute acid pretreatment of corn stover and its effect on pretreatment efficiency, inhibitor generation, sugar production, and bioconversion efficiency through simultaneous saccharification and ethanol fermentation (SSF) were evaluated.

**Results:**

The overall cellulose conversion taking account of cellulose loss in pretreatment was used to evaluate the efficiency of pretreatment. The two-phase computational fluid dynamics (CFD) model on dry pretreatment was established and applied to analyze the mixing mechanism. The results showed that the pretreatment efficiency was significantly improved and the inhibitor generation was reduced by the helically agitated mixing, compared to the dry pretreatment without mixing: the ethanol titer and yield from cellulose in the SSF reached 56.20 g/L and 69.43% at the 30% solids loading and 15 FPU/DM cellulase dosage, respectively, corresponding to a 26.5% increase in ethanol titer and 17.2% increase in ethanol yield at the same fermentation conditions.

**Conclusions:**

The advantage of helically agitated mixing may provide a prototype of dry dilute acid pretreatment processing for future commercial-scale production of cellulosic ethanol.

## Background

Pretreatment is the crucial step to overcome the biorecalcitrance of lignocellulose to achieve efficient bioconversion of cellulose into fermentable sugars and then to fermentation products such as ethanol [1-6]. Among various pretreatment methods, dilute sulfuric acid pretreatment is considered to be the one with potential commercial applications [7-12]. The major disadvantages of dilute acid pretreatment include relatively massive acidic waste water generation caused by low solids (lignocellulosic feedstock) content, loss of fermentable sugars during the solids/liquid separation after pretreatment, and relatively high inhibitor compounds generation [13]. To overcome these disadvantages, recent studies on dilute acid pretreatment have tried to increase the feedstock content of lignocellulose solids as high as possible [14-16]. One example in our previous study was a dry dilute acid pretreatment of corn stover, in which the solids content in the pretreatment was fed to an extreme high of up to 70% of the total feedstock [17] and successfully applied to production of ethanol, lipid, and lactic acid from corn stover [18-21]. This dilute acid pretreatment was called a ‘dry’ method, because both the corn stover feedstock and the pretreated corn stover product were ‘dry’ with no free water generation during the pretreatment, while the inhibitor generation was kept at a low level. In this way, the three major disadvantages of dilute acid pretreatment could be overcome: dry-in and dry-out thus no waste water was generated, dry pretreated product thus no solids/liquid separation was needed, and low inhibitor generation maintained a high pretreatment efficiency.

The practice of dry dilute acid pretreatment operation revealed that well mixing of the majority of lignocellulose solids with minimum steam input in the pretreatment reactor was the major challenge. When the dilute acid pretreatment was operated under a low solids/liquid ratio, the mixing of hot steam with corn stover feedstock was relatively easy, because the steam heated the continuous water phase, then the hot liquid heated the solids particles impregnated in the liquid. However, when dilute acid pretreatment was operated under a high solids/liquid ratio, such as the ‘dry’ condition described above [17], the mixing of the hot steam with the dry solids particles, and the heat transfer from the hot steam to the solids feedstock became very difficult for three reasons: no aqueous phase existed as a continuous phase covering the solids bulk body (heat transfer directly occurred from the hot steam to the solid corn stover), lignocellulose biomass was typically a good insulator to reduce the heat transfer from the surface to the inside part (the surface of a paced pile of biomass was at target temperature but the core of the packed bed was below the desired temperature causing uneven heating), and the steam at a low usage (less than half of the solids used according to Zhang *et al*. [17]) had to reach the scattered solids particles directly.

On the other hand, since lignocellulose materials possessed high water or steam absorption capacity, the steam entering the pretreatment reactor was quickly absorbed by the lignocellulose materials close to the feeding nozzle regions and could not be dispersed onto the materials in the upper region of the reactor. In a small bench scale reactor, the reactor volume was small and the steam injection could penetrate through the relatively thin packing materials in both the height and diameter of the reactor. However, with the increased pretreatment scale of industrial reactors, enforced mixing is inevitably required because it is not possible to distribute the steam jetting uniformly into the large pretreatment reactors through the thick packing in meters of height and diameter.

The required agitation system should work well with a completely dry solids system at reasonable energy consumption. In our previous studies, the helical ribbon stirred agitation and was found to achieve a well mixing condition of the solids majority with the liquid (enzyme) minority in the simultaneous saccharification and fermentation (SSF) of various pretreated lignocellulose materials [20-22]. However, the difference of mixing scenarios between these fermentation bioreactors and the pretreatment reactor was that in bioreactors, mixing started with the solids feedstock but these solids quickly changed into the liquid slurry, while in the pretreatment reactor, the mixing apparatus had to face a completely solids phase throughout the whole operation time when the dry pretreatment system was applied.

In this work, the mixing performance of helically agitated mixing in the dry dilute acid pretreatment was investigated. The mixing effect by the helical ribbon stirrer was first tested in a mock-up experiment using three reactors of different sizes. In the base of the results of the mock-up experiment, a new pretreatment reactor equipped with a helical ribbon stirrer was designed and the mixing performance of the majority of corn stover solids with minimum steam input was tested. The pretreatment efficiency was evaluated by enzymatic hydrolysis and ethanol fermentation. These results all indicate that the helically agitated mixing worked well for the dry dilute acid pretreatment with the enhanced bioconversion efficiency of corn stover to ethanol. This study may provide a suitable prototype of a pretreatment reactor at high solids loading for future large-scale or industrial-scale pretreatment operations.

## Results and discussion

### Pretreatment performance in the reactors with and without mixing

**Figure 1:**
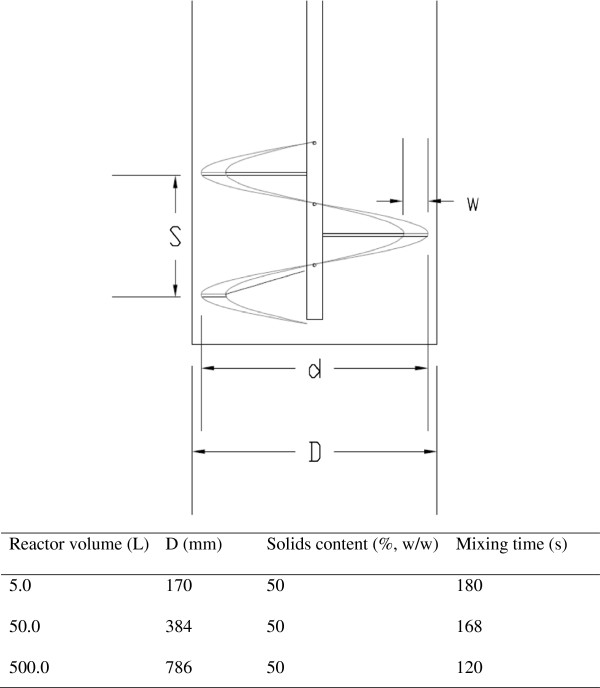
**Schematic diagram of the fluid dynamic reactors equipped with helical ribbon stirrer.** The reactor was a cylinder equipped with a helical ribbon stirrer. The parameters of the helical ribbon stirrer were matched with the change in the diameter of the reactor to keep the character of the helical ribbon the same in the three reactors. The mixing time was calculated by the time to reach constant moisture of corn stover in the reactors. d, diameter of impeller (mm); D, diameter of the reactor (mm); S, pitch size of the helical ribbon (mm); w, ribbon width (mm).

Helical screw feeders or conveyors are frequently used in lignocellulose processing plants as described in the National Renewable Energy Laboratory (NREL) technical report [12]. However, when the solids content gets very high, the compact screw device does not work well. Authors have tested several screw devices for mixing in the dry pretreatment reactor at corn stover solids of 70% (w/w), but the screw devices were damaged due to the high resistance to the screw movement (data not shown). Therefore, the loosely structured helical ribbon stirrer used in the previous SSF studies of pretreated corn stover at 30% (w/w) solids was tested. The fluid dynamic mock-up experiments at solids content of 50% (w/w) were carried out in several reactors of different sizes to test the mixing efficiency of corn stover with water. Figure 1 shows that the mixing of corn stover solids with water at solids content of 50% (w/w) was completed within 2 to 3 minutes. The positive fluid dynamic results plus the successful applications in high solids loading of SSF suggest that the helical ribbon stirrer might be a suitable agitation apparatus for dry pretreatment with very high solids content of up to 70% (w/w).

The dry dilute acid pretreatment of corn stover in both the helically agitated reactor and the static reactor (without mixing apparatus) was carried out and analyzed. In the previous study, the pretreatment reactor was a 10 L stainless cylinder of 180 mm in diameter and 400 mm in height [17]. Three steam injection nozzles in the bottom of the reactor were relatively sufficient to mix the steam with the lignocellulose materials packed inside the reactor. In this study, the pretreatment reactor was enlarged in diameter from 180 mm to 260 mm and the volume was increased from 10 L to 20 L (the height was kept unchanged at 400 mm), but the number and size of the steam injection nozzles were still the same. The purpose of the new enlarged reactor design was to demonstrate a scale-down example of the industrial pretreatment reactor in which the steam jetting was not efficiently mixed with the lignocellulose materials. The focus of this study is to find a solution for the problem in large-scale pretreatment reactors.

The original research plan on the enlarged pretreatment reactor was to operate the dry dilute acid pretreatment at exactly the same optimal conditions as that in the previous 10 L reactor: 190°C, 3 minutes, and 2.5% sulfide acid. Unfortunately, the new reactor (20 L) was twice as large as the previous one (10 L) and the agitation apparatus provided more heat dissipation. Thus, the steam supply from the same steam generator DZFZ4.5C was not sufficient for the new enlarged reactor and the maximum temperature was only 185°C, 5°C below the planned temperature (190°C).

On the other hand, the pretreatment efficiency in the new reactor at zero agitation was very poor, the inhomogeneity could be observed even with basic examination: large bulks of dark over-pretreated portions were at the bottom and yellow un-pretreated fresh portions were at the top. The main reasons were not only due to the enlarged diameter, which worsened the mixing of steam and lignocellulose materials, but also (and a major factor) due to the existence of the helical ribbon impeller parts, which severely disturbed the steam flow and created many dead zones inside the reactor. Unless the agitation apparatus was removed from the new enlarged reactor and left the new reactor as an empty cylinder, as the previous 10 L reactor for the static pretreatment operation (which was not possible to operate on the new reactor), the direct comparison of the pretreated materials obtained at the agitated condition and static condition did not accurately reflect the true situation.

Therefore, the authors decided not to compare the pretreated materials obtained from the same enlarged reactor at the agitated and static conditions. Instead, the pretreated materials for evaluation were chosen from the static pretreatment operation in the previous 10 L reactor at the optimal condition (190°C, 3 minutes, and 2.5% sulfide acid), and from the agitated pretreatment operation at the optimal conditions of the enlarged 20 L reactor (185°C, 3 minutes, and 2.5% sulfide acid), although there was 5°C difference in the pretreatment temperature.

**Figure 2:**
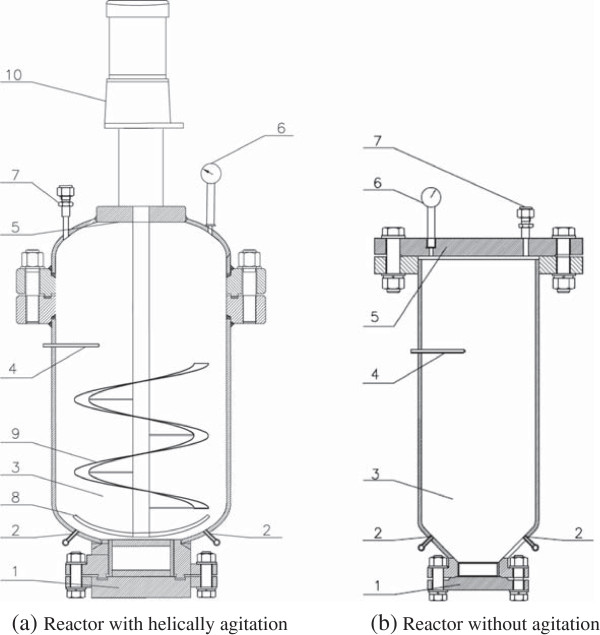
**Schematic diagram of the dry pretreatment reactors with and without helical agitation mechanism. (a)** Reactor equipped with helical ribbon impeller with the inner volume of 20 L; **(b)** Reactor without agitation apparatus with the inner volume of 10 L. 1, product outlet; 2, steam inlet; 3, pretreatment vessel; 4, thermocouple; 5, cap of the reactor; 6, pressure gauge; 7, inert air outlet; 8, anchor stirrer; 9, helical ribbon stirrer; 10, electric motor for driving the helical ribbon impeller.

Four operation cases are shown in Table 1. Case 1 and Case 2 were operated on the helically agitated reactor (Figure 2a) and lasted for 3 minutes at 185°C at different sulfuric acid usage: 2.0% (2.0 g per 100 g dry solids) for Case 1 and 2.5% for Case 2, respectively. Case 3 and Case 4 were operated on the static reactor without mixing apparatus (Figure 2b) at 190°C for 3 minutes at the same sulfuric acid usage of 2.0% and 2.5%, respectively. There was a temperature difference of 5°C between the two reactors, 185°C at Case 1 and Case 2, and 190°C for Case 3 and Case 4, because of the limitation of steam supply with the increased reactor size. However, the comparison among the operation cases of the two reactors still revealed sufficient information of the impact of helically agitated mixing on the dry pretreatment processing.

**Table 1:** Pretreatment performance in the static reactor and in the helically agitated reactor

**Pretreatment conditions**	**Cellulose conversion (%)**	**Inhibitors in the pretreated CS (g/100 g DM)**			**Sugars in the pretreated CS (g/100 g DM)**			
		**Furfural**	**5-HMF**	**Acetate**	**Glucose**	**Xylose**	**O-Glu**	**O-Xyl**
In helically agitated reactor								
Case 1: 185°C, 2.0%, 3 minutes	77.55	0.18	0.09	0.58	0.48	5.34	0.72	8.45
Case 2: 185°C, 2.5%, 3 minutes	87.11	0.63	0.17	0.81	1.01	10.20	1.10	2.84
In no agitation reactor								
Case 3: 190°C, 2.0%, 3 minutes	72.10	0.50	0.25	0.77	0.88	5.58	2.27	7.55
Case 4: 190°C, 2.5%, 3 minutes	85.10	0.90	0.21	1.20	1.58	8.02	1.57	4.29

The data in the pretreatment conditions column indicate the pretreatment temperature, acid usage, and residual time, respectively. Experiments were carried out in duplicate and averaged to give the mean values. Enzymatic hydrolysis was carried out at 50°C, 15 FPU enzyme dosage, and 150 rpm agitation for 72 hours. 5-HMF, 5-hydroxymethylfurfural; CS, corn stover; DM, dry matter; FPU, filter paper unit; O-Glu, glucan oligomer; O-Xyl, xylan oligomer.

Table 1 indicates that the helically agitated mixing in the dry pretreatment played a crucial role in promoting the pretreatment efficiency and reducing the inhibitor generation. At the sulfuric acid usage of 2.0% for 3 minutes, the cellulose conversion increased from 72.10% (Case 3) to 77.55% (Case 1), while at the sulfuric acid usage of 2.5% for 3 minutes, the cellulose conversion increased from 85.10% (Case 4) to 87.11% (Case 2). It is worth stressing that the increase of cellulose conversion occurred with a 5°C lower temperature in the helically agitated reactor (185°C) than that in the static reactor (190°C). Generally, the lower temperature in the pretreatment operation leads to lower pretreatment efficiency [9,11,17,23,24]. However, the opposite results were obtained, in which pretreatment efficiency at the lower temperature under the agitated condition was elevated, compared to that under the static pretreatment condition. The result clearly confirmed the advantage of helically agitated mixing on the pretreatment efficiency.

Furthermore, Table 1 also indicates that the inhibitor concentration in the pretreated corn stover dramatically decreased in the helically agitated pretreatment, although generally the inhibitor concentration increased with increasing pretreatment efficiency [14,15,17,25]. The comparison of Case 1 and Case 3 indicates that the three major inhibitors, furfural, 5- hydroxymethylfurfural (5-HMF), and acetic acid, decreased from 0.50, 0.25, and 0.77 g/100 g dry matter (DM) in Case 3 (without mixing) to 0.18, 0.09, and 0.58 g/100 g DM in Case 1 (with helically mixing), respectively. Similarly, the comparison of Case 2 and Case 4 indicates that these inhibitors decreased from 0.90, 0.21, and 1.20 g/100 g DM in Case 4 (without mixing) to 0.63, 0.17, and 0.81 g/100 g DM in Case 2 (with helically mixing), respectively. Corresponding to the inhibitor generation, the glucose and its oligomer concentrations in the pretreated corn stover decreased in the helically agitated reactor, and the xylose and oligomer concentrations were kept approximately the same.

### CFD modeling of the helically agitated mixing in the dry pretreatment

The well mixing of corn stover solids with steam by helical agitation could lead to a uniform distribution of temperature and sulfuric acid concentration, thus overheating at the bottom or underheating near the top of the reactor could be avoided. To illustrate the effect of helical agitation on the dry pretreatment efficiency, the computational fluid dynamics (CFD) method was used to simulate the steam flow with the corn stover solids in the helical ribbon stirrer agitated reactor. A simplified CFD model was established under several assumptions and the fluid dynamic state of the helically agitated reactor was simulated.

**Figure 3:**
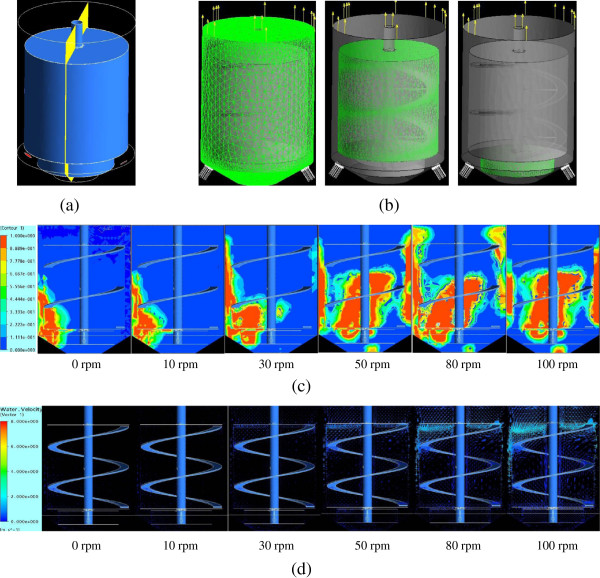
**CFD modeling of steam holdup and solids flow in the helically agitated pretreatment reactor. (a)** The reactor diagram in which the flow regime of the cross-section was simulated in the CFD calculation. **(b)** Geometric structure of the reactor in the CFD model. Left, mesh cells and structure; middle, motion region of the helical ribbon impeller; and right, motion region of the bottom anchor stirrer. **(c)** Conservative gas volume fraction under different agitation rates. **(d)** Fluid velocity distribution at different agitation rates. In this modeling, pretreated CS materials were assumed to the high viscose liquid with high apparent viscosity of 2.31 Pa · s; the hot steam stream was assumed to be inert air with a flow rate of 1.75 m/s. CFD, computational fluid dynamics; CS, corn stover.

Figure 3c indicates that the steam holdup (represented by the conservative gas volume fraction) was significantly improved by helical agitation. At the static state or the low agitation rate (0, 10, 30 rpm), the steam was accumulated to a very limited region near the jetting nozzles and the reactor walls, then quickly diffused upwardly without sufficient contact with solids. When the rotation rate was increased to 50 rpm, the steam holdup increased everywhere in the solids bulk, indicating that a well mass and heat transfer state was established.

Figure 3d also indicates that solids mixing (represented by the liquid velocity distribution) did not occur at no agitation or a low agitation rate in the reactor; with the increasing agitation rate, solids mixing was quickly improved and a reasonable fluid flow regime was established inside the reactor. On the other hand, Figure 3c and d reveal that the improvement of both steam holdup and solids mixing did not require a high agitation rate, thus an agitation rate of 50 rpm should be a suitable value for the present reactor.

The CFD modeling illustrated a relatively broad but clear picture of the improved mixing performance by helical agitation in the pretreatment reactor. The modeling results confirmed the estimation proposed at the beginning of this section.

### Improving pretreatment efficiency and reducing inhibitor generation by helically agitated mixing

**Figure 4:**
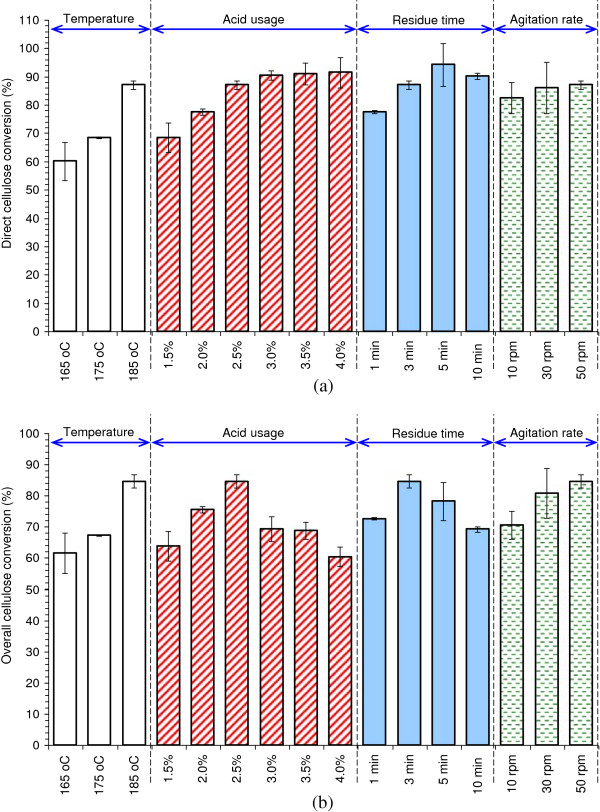
**Enzymatic hydrolysis assay of the pretreatment parameters in the helically agitated reactor. (a)** Direct cellulose conversion of the pretreated corn stover. **(b)** Overall cellulose conversion considering the cellulose recovery after the pretreatment. For detailed pretreatment operation, the experiment of changing temperature was carried out at 2.5% acid usage, 3 minutes of residue time, and 50 rpm agitation rate; the experiment of changing acid usage was carried out at 185°C, 3 minutes of residue time, and 50 rpm agitation rate; the experiment of changing residue time was carried out at 185°C, 2.5% acid usage, and 50 rpm agitation rate; and the experiment of changing agitation rate was carried out at 185°C, 2.5% acid usage, and 3 minutes of residue time. All the enzymatic hydrolysis processes of different pretreatment conditions were carried out at 5% solids loading (dry materials), 15 FPU/g DM cellulase dosage, pH 4.8, and 50°C. DM, dry matter; FPU, filter paper unit.

The dry pretreatment performance of corn stover in the helically agitated reactor was optimized by changing pretreatment parameters for maximum hydrolysis yield and minimum inhibitor generation. The results are shown in Tables 2 and 3 and Figure 4.

**Table 2:** Glucan and xylan recovery of the dry pretreatment in the helically agitated reactor

**Pretreatment conditions**	**Cellulose content before pretreatment (%)**	**Xylan content before pretreatment (%)**	**Glucan recovery (%)**	**Xylan recovery (%)**
Raw corn stover	37.15 ± 0.22	19.86 ± 0.56	-	-
Changing temperature				
**165°C** , 2.5%, 3 minutes	38.04 ± 2.04	8.57 ± 2.26	100.09	94.24
**175°C** , 2.5%, 3 minutes	37.23 ± 0.75	5.28 ± 0.02	96.69	79.32
**185°C** , 2.5%, 3 minutes	40.93 ± 0.06	2.93 ± 0.16	96.50	62.00
Changing acid usage				
185°C, **1.5%**, 3 minutes	37.71 ± 1.07	6.48 ± 0.18	92.05	78.89
185°C, **2.0%**, 3 minutes	37.41 ± 0.06	4.16 ± 0.08	96.63	74.91
185°C, **2.5%**, 3 minutes	40.93 ± 0.06	2.93 ± 0.16	96.50	62.00
185°C, **3.0%**, 3 minutes	38.48 ± 0.40	2.52 ± 0.17	73.97	43.18
185°C, **3.5%**, 3 minutes	36.85 ± 1.25	1.79 ± 0.21	75.12	36.37
185°C, **4.0%**, 3 minutes	35.98 ± 1.10	1.05 ± 0.10	65.53	29.75
Changing residue time				
185°C, 2.5%, **1 minute**	41.52 ± 5.88	4.06 ± 0.41	92.71	64.08
185°C, 2.5%, **3 minutes**	40.93 ± 0.06	2.93 ± 0.16	96.50	62.00
185°C, 2.5%, **5 minutes**	38.54 ± 1.88	2.93 ± 0.08	82.78	57.29
185°C, 2.5%, **10 minutes**	39.29 ± 0.75	2.21 ± 0.22	76.44	42.96
Changing agitation rate				
185°C, 2.5%, 3 minutes, **10 rpm**	40.23 ± 2.59	3.00 ± 0.19	84.81	54.03
185°C, 2.5%, 3 minutes, **30 rpm**	38.41 ± 0.58	2.86 ± 0.87	93.20	64.19
185°C, 2.5%, 3 minutes, **50 rpm**	40.93 ± 0.06	2.93 ± 0.16	96.50	62.00

**Table 3:** Impact of the operation parameters on the inhibitor and sugar level of the dry pretreatment in the helically agitated reactor

**Pretreatment conditions**	**Inhibitors in the pretreated CS (g/100 g DM)**			**Sugars in the pretreated CS (g/100 g DM)**			
	**Furfural**	**5-HMF**	**Acetic acid**	**Glucose**	**Xylose**	**O-Glu**	**O-Xyl**
Changing temperature							
**165°C**, 2.5%, 3 minutes, 50 rpm	0.13 ± 0.02	0.03 ± 0.00	0.45 ± 0.15	0.43 ± 0.01	7.58 ± 0.14	0.94 ± 0.06	9.28 ± 1.11
**175°C**, 2.5%, 3 minutes, 50 rpm	0.24 ± 0.02	0.06 ± 0.00	0.55 ± 0.01	0.60 ± 0.01	10.05 ± 0.21	0.78 ± 0.03	6.42 ± 0.09
**185°C**, 2.5%, 3 minutes, 50 rpm	0.63 ± 0.01	0.17 ± 0.01	0.81 ± 0.15	1.01 ± 0.02	10.20 ± 0.02	1.10 ± 0.44	2.84 ± 0.45
Changing acid concentration							
185°C, **1.5%**, 3 minutes, 50 rpm	0.08 ± 0.01	0.04 ± 0.02	0.59 ± 0.06	0.45 ± 0.05	2.39 ± 0.00	0.72 ± 0.04	11.02 ±0.02
185°C, **2.0%**, 3 minutes, 50 rpm	0.18 ± 0.06	0.09 ± 0.07	0.58 ± 0.02	0.48 ± 0.00	5.34 ± 0.01	0.77 ± 0.00	8.45 ± 0.38
185°C, **2.5%**, 3 minutes, 50 rpm	0.63 ± 0.01	0.17 ± 0.01	0.81 ± 0.15	1.01 ± 0.02	10.20 ± 0.02	1.10 ± 0.44	2.84 ± 0.45
185°C, **3.0%**, 3 minutes, 50 rpm	0.89 ± 0.03	0.30 ± 0.03	1.29 ± 0.03	2.41 ± 0.02	9.99 ± 0.11	0.39 ± 0.01	0.81 ± 0.15
185°C, **3.5%**, 3 minutes, 50 rpm	0.78 ± 0.02	0.36 ± 0.07	1.24 ± 0.05	2.07 ± 0.02	7.85 ± 0.08	0.45 ± 0.01	0.80 ± 0.10
185°C, **4.0%**, 3 minutes, 50 rpm	0.84 ± 0.07	0.41 ± 0.17	1.22 ± 0.16	3.50 ± 0.02	8.34 ± 0.04	0.43 ± 0.07	0.45 ± 0.13
Changing residue time							
185°C, 2.5%, **1 minute**, 50 rpm	0.39 ± 0.03	0.12 ± 0.01	0.73 ± 0.04	0.79 ± 0.00	9.89 ± 0.03	0.90 ± 0.08	2.56 ± 0.35
185°C, 2.5%, **3 minutes**, 50 rpm	0.63 ± 0.01	0.17 ± 0.01	0.81 ± 0.15	1.01 ± 0.02	10.20 ± 0.02	1.10 ± 0.44	2.84 ± 0.45
185°C, 2.5%, **5 minutes**, 50 rpm	0.80 ± 0.15	0.27 ± 0.03	1.09 ± 0.07	1.21 ± 0.02	7.91 ± 0.06	0.96 ± 0.11	1.35 ± 0.04
185°C, 2.5%, **10 minutes**, 50 rpm	0.99 ± 0.10	0.30 ± 0.02	1.13 ± 0.07	1.17 ± 0.01	6.27 ± 0.07	0.69 ± 0.04	0.37 ± 0.08
Changing agitation rate							
185°C, 2.5%, 3 minutes, **10 rpm**	0.59 ± 0.04	0.18 ± 0.02	0.80 ± 0.10	0.99 ± 0.20	10.65 ± 0.35	0.49 ± 0.07	1.53 ± 0.00
185°C, 2.5%, 3 minutes, **30 rpm**	0.52 ± 0.02	0.19 ± 0.00	0.79 ± 0.11	0.97 ± 0.27	10.58 ± 0.23	0.78 ± 0.05	2.04 ± 0.04
185°C, 2.5%, 3 minutes, **50 rpm**	0.63 ± 0.01	0.17 ± 0.01	0.81 ± 0.15	1.01 ± 0.02	10.20 ± 0.02	1.10 ± 0.44	2.84 ± 0.45

The data in the pretreatment conditions column indicate the pretreatment temperature, acid concentration, and residual time, respectively. All the pretreatment experiments were carried out at 50 rpm. All the experiments were carried out in duplicate. Error was calculated as standard deviation. 5-HMF, 5-hydroxymethylfurfural; CS, corn stover; DM, dry matter; O-Glu, glucan oligomer; O-Xyl, xylan oligomer.

The data in the pretreatment conditions column indicate the pretreatment temperature, acid concentration, and residual time, respectively. Cellulose and xylan content were determined by two-step acid hydrolysis methods described in the Methods section. The recovery of cellulose and xylan was calculated by the ratio of cellulose and xylan content in the dry materials before and after pretreatment. Cellulose and xylan after pretreatment consisted of monosaccharides, oligosaccharides, glucan and xylan components, and furfural in the xylan recovery. All the experiments were carried out in duplicate and error was calculated as standard deviation except the recovery of cellulose and xylan, which was calculated with the total materials from two batches of pretreatment at the same condition.

Table 2 indicates that the cellulose content after the pretreatment was almost the same with the virgin corn stover, but the xylan content decreased sharply with increasing temperature and acid usage. Glucan recovery was almost constant with the increasing temperature in the experimental range (165 to 185°C), but suddenly decreased when the sulfuric acid usage was above 2.5% and the residue time was longer than 3 minutes. This result gave a strong indication that in the present helically agitated reactor, the acid concentration and long pretreatment time were not preferred because the cellulose was easily converted into other degradation compounds such as 5-HMF at such a condition. On the other hand, the xylan recovery was relatively low compared to the cellulose recovery and decreased steadily with the increasing intensity of temperature, acid usage, and residue time. As illustrated in Table 3, the concentrations of the typical inhibitors such as furfural, 5-HMF, and acetic acid increased with increasing pretreatment intensity; the glucose increased with the increasing intensity but the oligomer showed the opposite tendency; xylose increased with pretreatment intensity but when it was too strong, xylose and its oligomer decreased possibly due to the generation of its downstream products such as furfural. The results indicate that at the present pretreatment, xylan degradation was still strong and the pretreatment condition should be compromised by considering cellulose/hemicellulose loss, inhibitor generation, and cellulose conversion, instead of glucose yield only.

Figure 4a indicated the direct cellulose conversion of the pretreated corn stover, while Figure 4b indicated the overall cellulose conversion of the virgin corn stover with the consideration of solid weight loss in the pretreatment. Figure 4a shows that the direct cellulose conversion increased with increasing pretreatment temperature, acid usage, and residue time, which was also in agreement with the tendency of general dilute acid pretreatments. The direct cellulose conversion at different acid usage increased until the acid usage reached 3.0% and remained almost unchanged as the acid usage increased further. The same trend could be observed when the residue time was prolonged. On the other hand, the overall cellulose conversion increased with increasing temperature and also increased with increasing acid usage till 2.5%, then decreased with further increase of acid usage, because of the weight loss mentioned above. The residue time also showed the same tendency on the overall cellulose conversion. The direct cellulose conversion and the overall cellulose conversion of the pretreated corn stover gradually increased with the increasing agitation rate. The pretreated corn stover materials in the reactor were driven out by the constant agitation. Therefore a minimum agitation rate (50 rpm for the 20 L reactor) was maintained because a very low agitation rate was not sufficient to drive the corn stover materials completely out of the reactor, and then led to the loss of cellulose and xylan. The maximum overall cellulose conversion of 83.09% was observed at 185°C, 2.5% acid usage, and 3 minutes of residue time.

### Simultaneous saccharification and ethanol fermentation (SSF) of pretreated corn stover

**Figure 5:**
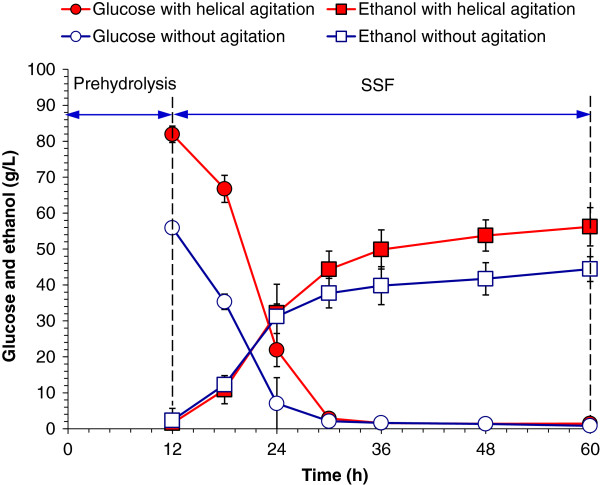
**SSF of the pretreated corn stover.** Corn stover was pretreated from the helically agitated reactor (185°C, 2.5%, 3 minutes, 50 rpm) and the no agitation reactor (190°C, 2.5%, 3 minutes), respectively. SSF was carried out under 30% solids loading, with the cellulase dosage of 15 FPU/g DM. The temperature and pH values during prehydrolysis process were controlled at 50°C and 4.8, respectively. These two conditions were set to 37°C and 5.5 during ethanol fermentation process by *Saccharomyces cerevisiae* DQ1, respectively. DM, dry matter; FPU, filter paper unit; SSF, simultaneous saccharification and fermentation.

The efficiency of the helically agitated pretreatment was tested by SSF using the dry dilute acid pretreated corn stover as feedstock. The corn stover was pretreated in the helically agitated reactor at 185°C for 3 minutes with 2.5% of sulfuric acid usage (Case 2 in Table 1); in the static reactor, the pretreatment was operated at 190°C for 3 minutes with 2.5% of sulfuric acid usage (Case 4 in Table 1). The pretreated corn stover was biodetoxified to remove the inhibitors until furfural and 5-HMF could not be detected. The SSF of the pretreated and detoxified corn stover was conducted under 30% solids loading (dry materials), 15 filter paper units (FPU)/g dry matter (DM) of cellulase dosage, and the results are shown in Figure 5.

Figure 5 shows that 12 hours’ prehydrolysis of corn stover after the helically agitated dry pretreatment released 81.92 g/L of glucose, and increased almost 47% compared to the glucose released from the hydrolysis of corn stover from the static dry pretreatment (55.87 g/L). The prehydrolysis results indicate that the pretreatment efficiency of corn stover from the helically agitated dry pretreatment was significantly improved. The SSF stage was started after 12 hours’ prehydrolysis and the significant improvement of ethanol yield was also observed: the ethanol titer reached 56.20 g/L after 48 hours’ SSF using the corn stover from the helically agitated pretreatment, while the ethanol titer was only 44.44 g/L under the same SSF conditions using corn stover from the static pretreatment. The ethanol yield from cellulose using the helically agitated pretreated corn stover was 69.34%, and the yield using statically pretreated corn stover was only 59.14%. The 26.5% and 17.2% increases of ethanol titer and yield were observed, respectively. The results indicated the advantage of the helically agitated well mixing in the dry pretreatment reactor.

The present ethanol titer of 56.20 g/L and yield of 69.43% were still not high enough because of the very high solids loading (30% w/w) and relatively short SSF time (48 hours). However, there is certainly sufficient space for improvement in ethanol titer, yield, and productivity of ethanol product. The helically agitated reactor in this study provided a prototype of dry dilute acid pretreatment processing under the output of no waste water generation, less sugar loss, low inhibitor generation, and low steam consumption.

## Conclusions

The helically agitated mixing significantly improved the efficiency of dry dilute acid pretreatment and reduced inhibitor generation compared to the dry pretreatment without agitation. For the dry dilute acid pretreatment at 70% solids loading of corn stover (dry base), an optimal pretreatment condition was obtained at 185°C, 2.5% of sulfuric acid usage, and lasted for 3 minutes. The ethanol titer and yield from cellulose in the SSF reached 56.20 g/L and 69.43% at 30% solids loading and 15 FPU/g DM cellulase, respectively, corresponding to 26.5% increase in the ethanol titer and 17.2% increase of ethanol yield at the same conditions. The advantage of helically agitated mixing in the dry pretreatment provided a prototype of dry dilute acid pretreatment for future commercial-scale production of cellulosic ethanol.

## Methods

### Raw materials

Corn stover was grown in Henan, China, and harvested in fall 2011. The corn stover materials were washed and then dried at 105°C until the weight was constant at which point the moisture was approximately 7% (w/w). The corn stover was then milled coarsely using a beater pulverizer (SF-300; Ketai Milling Equipment, Shanghai, China) and screened through a mesh with the circle diameter of 10 mm, then stored in sealed plastic bags until use.

### Strains and enzyme

*Amorphotheca resinae* ZN1 (stored at Chinese General Microorganisms Collection Center, Beijing, China; registration number: CGMCC 7452) was used as the biodetoxification strain for the removal of inhibitors from the pretreated corn stover [18]. *A. resinae* ZN1 was inoculated on the solids of pretreated corn stover which was neutralized with 20% Ca(OH)_2_ solution to pH 5.5. Biodetoxification started in solid state fermentation mode without any nutrients added and ended when the inhibitors were not detected on HPLC.

*Saccharomyces cerevisiae* DQ1 (stored at Chinese General Microorganisms Collection Center; registration number: CGMCC 2528) was used as the ethanol fermenting strain [22,26]. *S. cerevisiae* DQ1 was first cultured in the synthetic medium (20 g/L glucose, 2 g/L KH_2_PO_4_, 1 g/L (NH_4_)_2_SO_4_, 1 g/L MgSO_4_ · 7H_2_O, 1 g/L yeast extracts) for activation and transferred to the same medium without glucose containing the corn stover hydrolysate for adaption according to the procedure described by Zhang *et al*. [22].

The cellulase enzyme Youtell #6 was kindly provided by Hunan Youtell Biochemical Co. (Yueyang, Hunan, China). The filter paper activity of Youtell #6 was 135 FPU/g determined using the NREL Laboratory Analytical Procedure (LAP) LAP-006 [27], and the cellobiase activity was 344 cellobiase units (CBU)/g using the method of Sharma *et al*. [28].

### Fluid dynamic mock-up experiments

Mock-up experiments were designed to detect the mixing effect of corn stover and water by helical ribbon stirrer. The experiments were carried out in three reactors with different sizes of 5 L, 50 L, and 500 L. The inner structure of the three scales of reactor was the same, and detailed in Figure 1. Some parameters are also listed in the table below Figure 1. The mixing time of corn stover and water were used to illustrate the mixing effect. First, the corn stover was added into the reactor, and then the water was added from the inlet of the reactor into the corn stover when the agitation was turned on. The mixing time was calculated by the time to reach constant moisture of the corn stover.

### Pretreatment reactor

The detailed pretreatment reactor scheme is illustrated in Figure 2. Figure 2a shows the pretreatment reactor equipped with a helical ribbon stirrer. The reactor was a stainless cylinder with the working volume of 20 L (260 mm in diameter and 400 mm in height). The single helical ribbon stirrer was driven by a motor mounted on top of the reactor through an electromagnetic convertor. Figure 2b shows the pretreatment reactor with no mixing apparatus as a comparison. This reactor was previously used in Zhang *et al*. [17], and a stainless cylinder with a working volume of 10 L (180 mm in diameter and 400 mm in height) was also used. The hot steam was produced from the steam generator (DZFZ4.5C; Zhengyuan Electromechanics, Shanghai, China), then jetted into the reactor from the bottom and dispersed upward through several nozzles at the bottom. Two nozzles (6 mm in diameter) were designed on the distributor to disperse the steam jetted into the reactor at the mean steam flow rate of 0.1 to 1.0 kg per minute.

### Pretreatment operation

The dried corn stover was presoaked with the diluted sulfuric acid solution at the solid to liquid ratio of 2:1 (on the weight basis). The dilute acid solution was poured onto the dry corn stover materials then roughly mixed, and sealed in plastic bags and stayed for 12 hours at the ambient temperature (18 to 25°C). In each operation, 2,100 g of the presoaked corn stover (1,400 g of dry corn stover plus 700 g of dilute acid solution) was fed into the pretreatment reactor, and these corn stover materials roughly occupied the whole space of the reactor to meet the full solids loading condition of the reactor for reduction of steam consumption [17]. All the inlet valves were closed and the helical ribbon stirrer started to operate, then the steam valve was opened to jetting onto the presoaked corn stover. The purge valve was opened twice very briefly (2 to 3 seconds) to release the residual inert air inside the reactor. When the temperature reached the required value, the condition was maintained for a few minutes. To close the pretreatment operation, the steam supply was switched off and the steam inside the reactor was quickly released from the outlet of the reactor. The pretreated corn stover solids were taken out directly from the bottom of the reactor and no free water was released. Two batches of pretreatment at the same conditions were carried out, and the analysis of the pretreated corn stover was averaged from the two batches of pretreated corn stover.

### Pretreatment efficiency assay by enzymatic hydrolysis

The pretreated corn stover was assayed following the NREL LAP-009 [29]. One gram of the freshly pretreated corn stover (dry base) was added into 0.1 M citrate buffer (pH 4.8) to prepare the 5% (w/w) solids slurry in the flask. The cellulase dosage was 15 FPU/g DM (dry pretreated corn stover mass) and the hydrolysis lasted for 72 hours at 50°C and 150 rpm of shaking.

Cellulose and xylan recovery was calculated based on the dry weight of corn stover before and after pretreatment. Cellulose components after pretreatment included cellulose, glucose, and glucan oligomers in the dry materials; and xylan components included hemicellulose, xylose, and xylan oligomers in the dry materials. The recovery was defined as the ratio of cellulose and xylan content after pretreatment to those before pretreatment. The direct cellulose conversion of the pretreated corn stover was indicated by the ratio of the glucose produced after the 72 hours’ enzymatic hydrolysis (subtracting the initial glucose and glucan oligomers in the pretreated corn stover) to the theoretical glucose released from the cellulose in the pretreated corn stover. The overall cellulose conversion of corn stover was indicated by the ratio of the total glucose produced to the total theoretical glucose released from the original corn stover before pretreatment, in which the cellulose loss in the pretreatment was taken into account. The original cellulose content was calculated by the cellulose content of the pretreated corn stover divided by the cellulose recovery.

### Pretreatment assay by SSF

In the SSF process using the dry pretreated corn stover, the higher inhibitor concentration, which was caused by the high solids loading in the pretreatment, would greatly decrease the performance of the fermentative strains. Thus prior to the SSF step, the pretreated corn stover materials were detoxified biologically using the fungus *A. resinae* ZN1 according to the strictly uniformed procedure described in our reports [18,20,21] for all the pretreated SSF cases at the fixed time, temperature, and the operation. The SSF operation of the pretreated and biodetoxified corn stover was carried out in a 5 L helical ribbon stirrer agitated bioreactor as described in Zhang *et al*. [22]. The SSF operation was carried out at 30% solids (dry pretreated corn stover) concentration, 15 FPU/g DM cellulase dosage. The detoxified corn stover feedstock was sterilized at 115°C for 20 minutes. The operation started with 12 hours’ prehydrolysis at 50°C and pH 4.8, then the temperature was reduced to 37°C and the adapted *S. cerevisiae* DQ1 cells were inoculated into the bioreactor at 10% inoculum ratio (v/v) to start the SSF. Samples were taken periodically for analysis of ethanol and glucose. These experiments were carried out using the two separate batches of pretreated corn stover at the same pretreatment condition, and then averaged for the final data and error.

### Computational fluid dynamics (CFD) modeling of the pretreatment reactor

The commercial grid generation tool, ICEM CFD 11.0 (Ansys Inc., Canonsburg, PA, USA) was used to generate the three-dimensional grids of the reactor model created in SolidWorks 2010 (Dassault Inc., Vélizy-Villacoublay, France). The impeller agitation was characterized with the multiple reference frame (MRF) model. The mathematical model was solved in CFX 11.0 (Ansys Inc.). The initial and boundary conditions were specified as: 1) the impeller and shaft regions were stationary relative to the fluid domain; 2) no slip wall; 3) the residual error was set as 1 × 10^-4^; and 4) the Eulerian-Eulerian and the *k*-*ϵ* turbulence model were applied.

Figure 3a and b show the CFD mesh cells and geometric structure, respectively, in which the gas inlets were identical and the gas outlet was assumed to be released to the completely open cap on top of the reactor. A gas–liquid two-phase flow was assumed under the assumptions of the pseudo-liquid phase of corn stover materials, and the inert and incompressible gas phase of steam vapor. The apparent viscosity of the assumed pseudo-liquid was set to 2.31 Pa · s according to the determination using the torque measurement method. The density of the assumed gas flow was set to 14.18 g/L, which equaled the steam density at 3.0 MPa, 250°C used in the pretreatment. The velocity of the gas phase was calculated to be 1.75 m/s by modeling the typical pretreatment process, which equaled to jetting 700 g of hot steam within 3 minutes into the reactor through two nozzles. The conservative gas volume fraction and the liquid velocity distribution were used to characterize the mixing at different agitation conditions.

### Sugar and inhibitor analysis

Sugars and inhibitors were measured by HPLC (LC-20 AD, RID-10A refractive index detector; Shimadzu, Kyoto, Japan) equipped with an Aminex HPX-87H column (Bio-Rad, Hercules, CA, USA) at the column temperature 65°C. The mobile phase was 5 mM H_2_SO_4_ at the rate of 0.6 mL/min. Samples were filtered through a 0.22 μm membrane before analysis.

### Cellulose and xylan composition determination

The cellulose and xylan content of corn stover were measured by two-step acid hydrolysis according to NREL LAPs [30,31]. The pretreated corn stover was washed thoroughly with deionized water and oven dried at 105°C overnight to determine the content of water insoluble solids. One hundred milligrams of dried corn stover was added to 1 mL 72% (w/w) H_2_SO_4_ and then incubated at 30°C in a water bath for 1 hour with stirring by glass rod. The mixture was diluted to 29 mL in volume and hydrolyzed at 121°C for 1 hour. The hydrolyzed mixture was neutralized using CaCO_3_ powder and centrifuged. The supernatant was used for HPLC analysis to measure the glucose and xylose to calculate the cellulose and xylan content.

Oligomers of cellulose and xylan were measured according to NREL LAP [31]. The mixture of 5 g wet pretreated corn stover and 50 mL deionized water was shaken at 180 rpm for 2 hours at 30°C. Then 5 mL filtrate after solids/liquid separation was used for determining the concentration of glucose and xylose, and mixed with 1 mL 72% (w/w) sulfuric acid. The mixture was then diluted to 29 mL in volume and the subsequent process was the same as the two-step hydrolysis. The difference of the sugar concentration before and after acid hydrolysis was calculated as the oligosaccharide content. These experiments were carried out using the two separate batches of pretreated corn stover at the same pretreatment condition, and then averaged for the final data and error.

## Abbreviations

5-HMF: 5-Hydroxymethylfurfural; CBU: Cellobiase unit; CFD: Computational fluid dynamics; CS: Corn stover; DM: Dry matter; FPU: Filter paper unit; HPLC: High performance liquid chromatography; LAP: Laboratory Analytical Procedure; MRF: Multiple reference frame; NREL: National Renewable Energy Laboratory; O-Glu: Glucan oligomer; O-Xyl: Xylan oligomer; SSF: Simultaneous saccharification and fermentation.

## Competing interests

The authors declare that they have no competing interests.

## Authors’ contributions

YQH and JB designed the experiment and drafted the manuscript. JZ designed the equipment. YQH and JZ carried out the experiment. LPZ carried out the CFD calculation. JB conceived of the study. All authors read and approved the final manuscript.

## References

[B1] Wyman CE Dale BE Elander RT Holtzapple M Ladisch MR Lee YY Coordinated development of leading biomass pretreatment technologies Bioresour Technol 2005 96 1959 1966 10.1016/j.biortech.2005.01.010 16112483

[B2] Yang B Wyman CE Pretreatment: the key to unlocking low-cost cellulosic ethanol Biofuel Bioprod Bioref 2008 2 26 40 10.1002/bbb.49

[B3] Zhu JY Pan XJ Zalesny RS Jr Pretreatment of woody biomass for biofuel production: energy efficiency, technologies, and recalcitrance Appl Microbiol Biotechnol 2010 87 847 857 10.1007/s00253-010-2654-8 20473606

[B4] Alvira P Tomas-Pejo E Ballesteros M Negro MJ Pretreatment technologies for an efficient bioethanol production process based on enzymatic hydrolysis: a review Bioresour Technol 2010 101 4851 4861 10.1016/j.biortech.2009.11.093 20042329

[B5] Chiaramonti D Prussi M Ferrero S Oriani L Ottonello P Torre P Cherchi F Review of pretreatment processes for lignocellulosic ethanol production, and development of an innovative method Biomass Bioenergy 2012 46 25 35

[B6] Galbe M Zacchi G Pretreatment: the key to efficient utilization of lignocellulosic materials Biomass Bioenergy 2012 46 70 78

[B7] Torget R Werdene P Himmel M Grohmann K Dilute acid pretreatment of short rotation woody and herbaceous crops Appl Biochem Biotechnol 1990 24 115 126

[B8] Torget R Werdene P Himmel M Grohmann K Dilute-acid pretreatment of corn residues and short-rotation woody crops Appl Biochem Biotechnol 1991 28 75 86

[B9] Saha BC Iten LB Cotta MA Wu YV Dilute acid pretreatment, enzymatic saccharification and fermentation of wheat straw to ethanol Process Biochem 2005 40 3693 3700 10.1016/j.procbio.2005.04.006 15932261

[B10] Lloyd TA Wyman CE Combined sugar yields for dilute sulfuric acid pretreatment of corn stover followed by enzymatic hydrolysis of the remaining solids Bioresour Technol 2005 96 1967 1977 10.1016/j.biortech.2005.01.011 16112484

[B11] Hsu TC Guo GL Chen WH Hwang WS Effect of dilute acid pretreatment of rice straw on structural properties and enzymatic hydrolysis Bioresour Technol 2010 101 4907 4913 10.1016/j.biortech.2009.10.009 19926476

[B12] Humbird D Davis R Tao L Kinchin C Hsu D Aden A Schoen P Lukas J Olthof B Worley M Sexton D Dudgeon D Process Design and Economics for Biochemical Conversion of Lignocellulosic Biomass to Ethanol Technical Report NREL/TP-5100-47764 2011 NREL: Golden, CO

[B13] Dong HW Bao J Biofuel via biodetoxification Nat Chem Biol 2010 6 316 318 10.1038/nchembio.355 20404819

[B14] Linde M Jakobsson EL Galbe M Zacchi G Steam pretreatment of dilute H_2_SO_4_-impregnated wheat straw and SSF with low yeast and enzyme loadings for bioethanol production Biomass Bioenergy 2008 32 326 332 10.1016/j.biombioe.2007.09.013

[B15] Sassner P Martensson GG Galbe M Zacchi G Steam pretreatment of H_2_SO_4_-impregnated Salix for the production of bioethanol Bioresour Technol 2008 99 137 145 10.1016/j.biortech.2006.11.039 17223555

[B16] Modenbach AA Nokes SE The use of high-solids loadings in biomass pretreatment—a review Biotechnol Bioeng 2012 109 1430 1442 10.1002/bit.24464 22359283

[B17] Zhang J Wang XS Chu DQ He YQ Bao J Dry pretreatment of lignocellulose with extremely low steam and water usage for bioethanol production Bioresour Technol 2011 102 4480 4488 10.1016/j.biortech.2011.01.005 21277774

[B18] Zhang J Zhu ZN Wang XF Wang N Wang W Bao J Biodetoxification of toxins generated from lignocellulose pretreatment using a newly isolated fungus *Amorphotheca resinae* ZN1 and the consequent ethanol fermentation Biotechnol Biofuels 2010 3 26 10.1186/1754-6834-3-26 21092158 PMC2998489

[B19] Huang X Wang YM Liu W Bao J Biological removal of inhibitors leads to the improved lipid production in the lipid fermentation of corn stover hydrolysate by *Trichosporon cutaneum* Bioresour Technol 2011 102 9705 9709 10.1016/j.biortech.2011.08.024 21880481

[B20] Liu W Wang YM Yu ZC Bao J Simultaneous saccharification and microbial lipid fermentation of corn stover by oleaginous yeast *Trichosporon cutaneum* Bioresour Technol 2012 118 13 18 22695140 10.1016/j.biortech.2012.05.038

[B21] Zhao K Qiao QA Chu DQ Gu HQ Dao TH Zhang J Bao J Simultaneous saccharification and high titer lactic acid fermentation of corn stover using a newly isolated lactic acid bacterium *Pediococcus acidilactici* DQ2 Bioresour Technol 2013 135 481 489 23127836 10.1016/j.biortech.2012.09.063

[B22] Zhang J Chu DQ Huang J Yu ZC Dai GC Bao J Simultaneous saccharification and ethanol fermentation at high corn stover solids loading in a helical stirring bioreactor Biotechnol Bioeng 2010 105 718 728 19882718 10.1002/bit.22593

[B23] Jensen JR Morinelly JE Gossen KR Brodeur-Campbell MJ Shonnard DR Effects of dilute acid pretreatment conditions on enzymatic hydrolysis monomer and oligomer sugar yields for aspen, balsam, and switchgrass Bioresour Technol 2010 101 2317 2325 10.1016/j.biortech.2009.11.038 20018506

[B24] Jung YH Kim IJ Kim HK Kim KH Dilute acid pretreatment of lignocellulose for whole slurry ethanol fermentation Bioresour Technol 2013 132 109 114 23395763 10.1016/j.biortech.2012.12.151

[B25] Redding AP Wang ZY Keshwani RD Cheng JJ High temperature dilute acid pretreatment of coastal Bermuda grass for enzymatic hydrolysis Bioresour Technol 2011 102 1415 1424 10.1016/j.biortech.2010.09.053 20943378

[B26] Chu DQ Zhang J Bao J Simultaneous saccharification and ethanol fermentation of corn stover at high temperature and high solids loading by a thermotolerant strain *Saccharomyces cerevisiae* DQ1 Bioenerg Res 2012 5 1020 1026 10.1007/s12155-012-9219-x

[B27] Adney B Baker J Measurement of Cellulase Activities Laboratory Analytical Procedure (LAP). LAP-006 1996 NREL: Golden, CO

[B28] Sharma S Sandhu DK Bagga PS Physical characterization of isozymes of endo-beta-1,4-glucanase and beta-1,4-glucosidase from *Aspergillus* species FEMS Microbiol Lett 1991 63 99 104 2044945 10.1016/0378-1097(91)90535-i

[B29] Brown L Torget R Enzymatic Saccharification of Lignocellulosic Biomass. Laboratory Analytical Procedure (LAP). LAP-009 1996 NREL: Golden, CO

[B30] Sluiter A Hames B Ruiz R Scarlata C Sluiter J Templeton D Crocker D Determination of Structural Carbohydrates and Lignin in Biomass Laboratory Analytical Procedure (LAP). Technical Report NREL/TP-510-42618 2008 NREL: Golden, CO

[B31] Sluiter A Hames B Ruiz R Scarlata C Sluiter J Templeton D Determination of Sugars, Byproducts, and Degradation Products in Liquid Fraction Process Samples Laboratory Analytical Procedure (LAP). Technical Report NREL/TP-510-42623 2008 NREL: Golden, CO

